# The Heart and Seizures: Friends or Enemies?

**DOI:** 10.3390/jcm12185805

**Published:** 2023-09-06

**Authors:** Elena Pasini, Roberto Michelucci

**Affiliations:** IRCCS Istituto delle Scienze Neurologiche di Bologna, Unit of Neurology, Bellaria Hospital, 40139 Bologna, Italy; roberto.michelucci@isnb.it

**Keywords:** epilepsy, syncope, ictal asystole, arrhythmia, loss of consciousness, SUDEP

## Abstract

The heart and seizures are closely linked by an indissoluble relationship that finds its basis in the cerebral limbic circuit whose mechanisms remain largely obscure. The differential diagnosis between seizures and syncopes has always been a cornerstone of the collaboration between cardiologists and neurologists and is renewed as a field of great interest for multidisciplinary collaboration in the era of the diffusion of prolonged telemonitoring units. The occurrence of ictal or post-ictal arrhythmias is currently a cause of great scientific debate with respect to the role and risks that these complications can generate (including sudden unexpected death in epilepsy). Furthermore, the study of epileptic seizures and the arrhythmological complications they cause (during and after seizures) also allows us to unravel the mechanisms that link them. Finally, intercritical arrhythmias may represent great potential in terms of the prevention of cardiological risk in epileptic patients as well as in the possible prediction of the seizures themselves. In this paper, we review the pertaining literature on this subject and propose a scheme of classification of the cases of arrhythmia temporally connected to seizures.

## 1. Introduction

Since ancient times, physicians have been fascinated by the complex and heterogeneous interaction between the heart and the brain, establishing in different epochs the supremacy of one or the other in the control of emotions. If in the past (in the Egyptian period as well as at the time of the great philosopher Aristotele) the cardiocentric model reigned (the Latin *recordor*—to remember—and its opposite *obliviscor*—to forget—both contain the root *cor* identifying the heart as a container of memory), modern history has appointed the brain as the seat of feelings even though referring to its most ancient and primitive part: the limbic system. The latter has been a source of great interest for the study of all neurological diseases as it has direct control of the bidirectional interaction between emotions and the autonomic system, i.e., between the brain and the heart.

The limbic system is composed of cortical and subcortical structures (the cingulate gyrus, parahippocampal gyrus, hippocampal formation, dentate gyrus, hippocampus, subicular complex, amygdala, septal area, and hypothalamus).

These structures form a complex network for controlling emotion, which can obviously, in turn, influence hypothalamic autonomic responses, inducing respiratory, blood pressure, and heart rate changes. On the other side, autonomic changes are known to influence homeostasis, and via the posterior cortex of the insula, this would mediates unconscious changes in behavior.

Loss of consciousness, in particular, represents the clinical synthesis of all the pathological interactions between the heart and the brain induced by the limbic system, spanning from the situational syncope caused by pain or fear to focal epileptic seizures with secondary tonic–clonic evolution started by anguish and tachycardia.

The relationships between syncopes and seizures are complex and reciprocal. While in most cases it is a matter of differential diagnosis, in rare cases, a seizure may cause a syncope or, alternatively, a syncope may trigger a seizure.

The routine use of video-EEG recording in the emergency department or the widespread use of long-term video-EEG monitoring in epilepsy units coupled with EKG has allowed us to “capture” these episodes and to diagnose concomitant cardiogenic events related to seizures. The discovery of ictal or post-ictal arrhythmic events (ranging from asystole to atrial or ventricular fibrillation) has raised the suspicion that they could be responsible for sudden unexpected death in epilepsy (SUDEP) and has stimulated wide research in this field. On the other hand, it is well known that specific mutations of genes modulating ion channels are responsible for arrhythmogenic cardiopathies and some forms of epilepsy, giving a clue to a possible pathogenetic mechanism.

These heart–brain relationships in epilepsy increase interest in the field as well because they enable research on automatic detectors of seizures. The major problem of epileptic patients in fact lies in the impossibility of predicting the occurrence of seizures. A number of studies on heart rate variability (HRV) have documented its relevance. Modifications of HRV before seizures seem to predict the ictal event besides giving important information about the state of the vegetative system of patients with epilepsy.

In this paper, we review the pertaining literature on this subject and also propose a scheme of classification of these cases of arrhythmia temporally connected to seizures.

## 2. Methods

### 2.1. Search of the Literature

We performed a broad literature search on the PubMed database for studies investigating the association between arrhythmias and epilepsy. We searched for studies published up to 1 June 2023 without backward limits, using the following MeSH: “arrhythmia” AND “seizure”. Subsequently, we searched for “seizure” AND one of the following terms: “asystole”, “bradycardia”, “tachycardia”, “atrial fibrillation”, “ventricular fibrillation”, “atrio-ventricular block”, and “Takotsubo Syndrome”. To avoid the potential omission of relevant studies, we also manually screened the reference list of papers included and previous reviews regarding similar topics. Duplicate articles were eliminated manually. 

### 2.2. Study Selection

The research strategy relied on title and abstract analysis. An article’s full text was retrieved if the title and abstract met the inclusion criteria. No automatic tools were used in this phase.

### 2.3. Inclusion Criteria

We included in our selection only documented case reports coupled with documentation of the seizure or arrhythmia; only English-language studies were considered. 

### 2.4. Qualitative Data Extraction

According to the above-mentioned criteria, all articles were screened and identified by both authors. The extracted qualitative data included the following: author, publication year, aim of the study, and principal findings.

## 3. Clinical Cardiovascular Events Connected to Seizures

### 3.1. Ictal Arrhythmias

Bradycardia and tachycardia. Heart rate (HR) modifications are very common during focal seizures, in particular those arising from the temporal lobe where they are estimated to occur in almost all cases [1]. 

**Figure 1:**
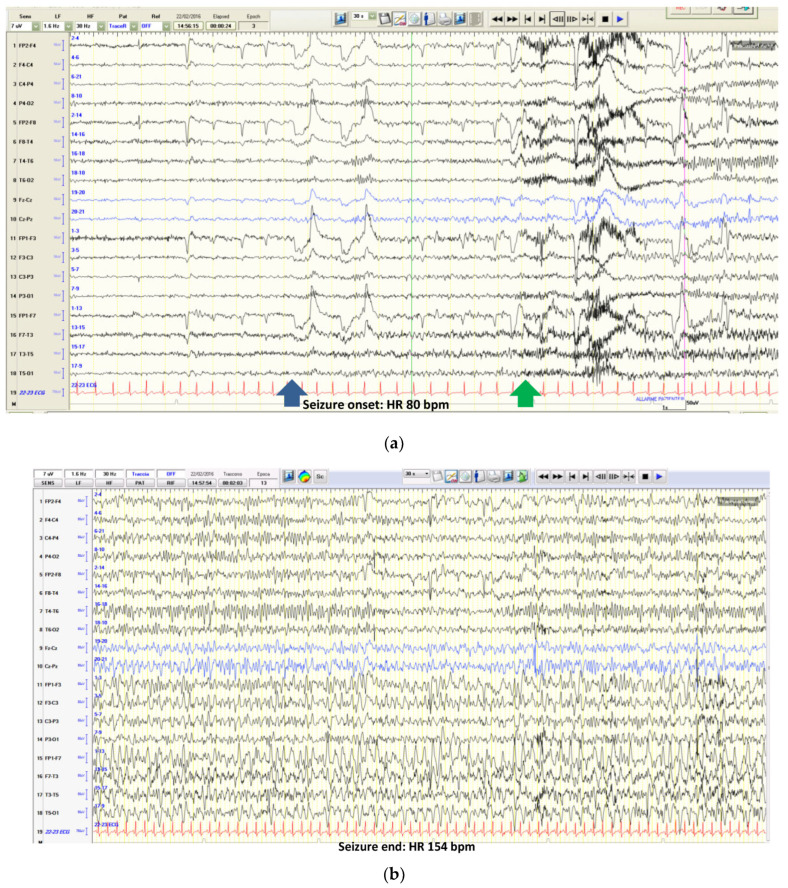
(**a**): The pre-ictal HR was 80 bpm. Seizure onset is evident in the frontotemporal region with a rhythmic theta activity (see the blue arrow). HR begins to increase (see the green arrow). (**b**): During the same seizure (strictly confined to left frontotemporal regions and in the absence of motor involvement), HR progressively increases to 154 bpm (nearly 200% more).

Tachycardia is by far the most common phenomenon and it is reported to occur in over 82% of cases, generally during the first 30 s from seizure onset, inducing more than 30 bpm of change in HR. Intra-individual variability in terms of HR increase or HR increase progression during a seizure has been described in several papers [2] (see an example of tachycardia during a focal left frontotemporal seizure in Figure 1a,b).

On the other hand, cardiac rhythm changes like bradycardia or asystole that occur during some seizures are less common but probably underestimated. In large epilepsy centers, ictal cardiac asystole was reported in 0.1—0.4% of patients undergoing video-EEG monitoring [1,2,3,4,5,6,7].

In other studies, however, the percentage was higher, up to 16% of recorded seizures [8]. In most reported cases, ictal bradycardia and asystole (IA) were associated with focal seizures originating from the temporal lobe with only a slight left predominance [2,3,4,5,6,7,9]. Interestingly, a few cases of IA recorded during an SEEG recording showed an insular origin of the responsible seizures, confirming the involvement of the limbic system [10,11].

**Figure 2:**
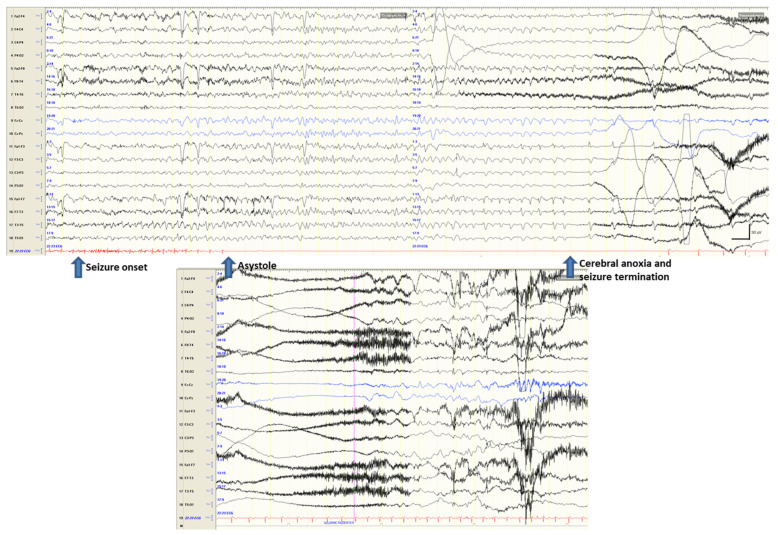
Seizure onset from the left temporal region is followed (after about 8 s) by ictal asystole. During the 24 s of asystole, the EEG traces show the signs of hypoperfusion with progressive slowing of activity evolving into EEG suppression (with seizure termination after 19 s since asystole onset).

Seizure-induced ictal asystole and syncopes. Over the years, a conspicuous number of IA cases have been reported whose electro-clinical symptoms have been fully described [12,13,14]. In detail, in 2017, Tenyi et al. [14] reviewed the available literature and quoted 157 cases of IA. Since then, 30 additional cases have been reported. In these cases, IA appears a few seconds after seizure onset and may be accompanied by syncope-like symptoms (falling to the ground and hypoperfusion signs) and EEG changes typical of syncope (see an example in Figure 2).

It is not yet clear how the delay between seizure onset and asystole might explain or influence the development of the symptomatology, while two clinical scenarios may be encountered [14].

The first and less common picture is the appearance of IA asystole in the context of new-onset/newly diagnosed epilepsy. In these cases, the attacks resemble syncopes, but their epileptic nature may be suggested by specific “epileptic auras” preceding the loss of consciousness (if present), the cluster occurrence of events, the absence of evident cardiogenic or situational triggers, and, in particular, an ictal EEG–EKG recording performed in the emergency room. Thirty-two patients described in 22 papers showed this specific presentation. The asystole was self-limiting in every case and lasted 20 s on average. The mean age at presentation was 46 years (ranging from 2 to 83 years; median: 47 years) with a clear female prevalence. In five cases, interictal alterations of the basal EKG were described, and this could be identified as a further risk factor. The majority of patients had frontotemporal epilepsy with a slight prevalence of left-side involvement (19 out of 31 cases), generally drug-responsive. Interestingly, in two patients, the IA was observed in the context of anti-NMDA receptor encephalitis [15,16], a condition that involves more generalized autonomic dysregulation.

The second and more common scenario consists of patients with a relatively long history of known focal (usually temporal) epilepsy who develop a change of seizure semiology with falls and syncope-like attacks and yet preceded by the usual epileptic auras. Around 160 patients belonging to 35 papers have been described in detail. In the study of Nguyen [17], reporting on nine patients, it was clear that the IA could be associated with a syncope-like EEG picture and syncopal symptoms when the duration of asystole was longer than 6 s (mean 16 s) while it did not cause any EEG change or symptoms when it was shorter (mean 6 s). Similar data were described by Bestaworos [18] in 10 cases, who confirmed that only IA > 6 s was strongly associated with ictal syncope. In the paper by Rubboli et al. [19], it was observed, however, that the patient’s fall to the ground occurred in association with short asystoles (6 s) without any syncope-like EEG changes. This finding raises the issue that hypoperfusion is not the only mechanism causing atonic seizures. Probably the progressive involvement of a diffuse epileptogenic network is the basis for the change in clinical symptomatology in these cases. 

Syncope-induced seizures. The reverse situation in which a vasovagal syncope can precipitate an epileptic seizure has only rarely been described and exceptionally documented on EEG recordings. Most cases concern children who have had generalized epileptic seizures associated with their syncopal events. Some patients had absence seizures [20] or generalized convulsive seizures, either clonic or tonic–clonic [21]. These seizures, also known as anoxic epileptic seizures, were recorded during a variety of syncopes, ranging from reflex asystolic, prolonged expiratory apnoea (cyanotic breath-holding spells), compulsive Valsalva maneuvers, or of mixed or uncertain origin. A case of atypical seizure with mesial temporal onset induced by a vaso-vagal syncope was recorded during SEEG in an adult patient with refractory focal epilepsy [22].

**Figure 3:**
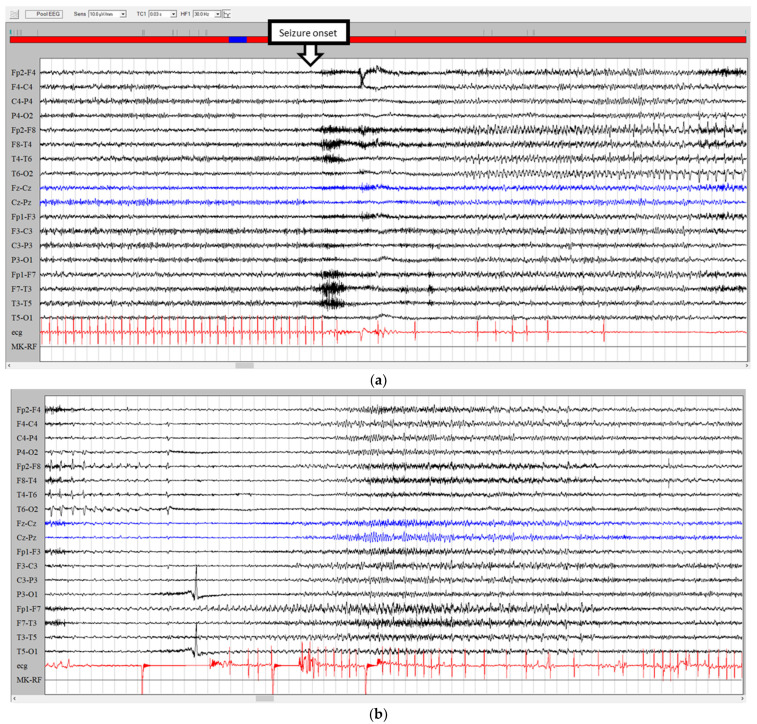
(**a**) The EEG traces show a right frontotemporal recruiting activity rapidly associated with ictal arrhythmia (with a 28 s asystole). (**b**) The right ictal activity slows down as attended in the course of hypoxic attenuation of the tracing but unexpectedly restarts over the contralateral frontotemporal areas during asystole.

Overall, in these cases, which are probably underdiagnosed or misdiagnosed as pure syncopes or seizures, it is believed that transient anoxia caused by the syncope may trigger a seizure. Recently, a patient was reported as showing ictal asystole induced by a right-sided focal seizure precipitating in the same attack a contralateral electro-clinical focal seizure [23] (Figure 3). This evidence further complicates the scenario.

### 3.2. Other Ictal or Post-Ictal Arrhythmias

Atrial fibrillation (AF). Nineteen cases of peri-ictal atrial fibrillation have been described up to now. In the vast majority of cases, AF occurred as a post-ictal phenomenon, whereas in only two cases, a paroxysmal self-limiting arrhythmia developed during the ictal phase of a left limbic seizure [24].

Atrioventricular block (AV block). An increased vagal outflow responsible for IA might also explain the ictal AV block. Allana et al. 2014 [25] reported an interesting case of ictal bradycardia evolving into an AV block during a left limbic seizure. In particular, in this case, the cardiac rhythm analysis revealed, during progressive bradycardia, a transition from a normal sinus rhythm to an atrial-paced rhythm.

Ventricular fibrillation. A few cases of ventricular fibrillation induced by seizures have been reported so far, usually occurring in the post-ictal phase of focal seizures, evolving into bilateral tonic–clonic seizures, and causing a near-SUDEP [26]. Fortunately, the latter cases occurred in monitoring units, allowing for immediate treatment by means of cardiopulmonary resuscitation. Obviously in other settings (first of all at home), it may be fatal. 

### 3.3. Other Seizure-Related Cardiovascular Effects

Takotsubo Syndrome (TTS): This rare syndrome (also called stress cardiomyopathy, apical ballooning syndrome, or broken heart syndrome) was first described in Japan in 1990 in association with cerebral hemorrhages possibly causing a catecholamine storm. TTS predominantly affects elderly women and is often preceded by emotional or physical stress. The diagnosis stands on seven diagnostic criteria which include anatomical features, ECG changes, cardiac biomarkers, and the reversibility of myocardial dysfunction (see Table 1) [27]. 

Epileptic seizures as triggering events of TTS have been described in >74 cases [28].

**Figure 4:**
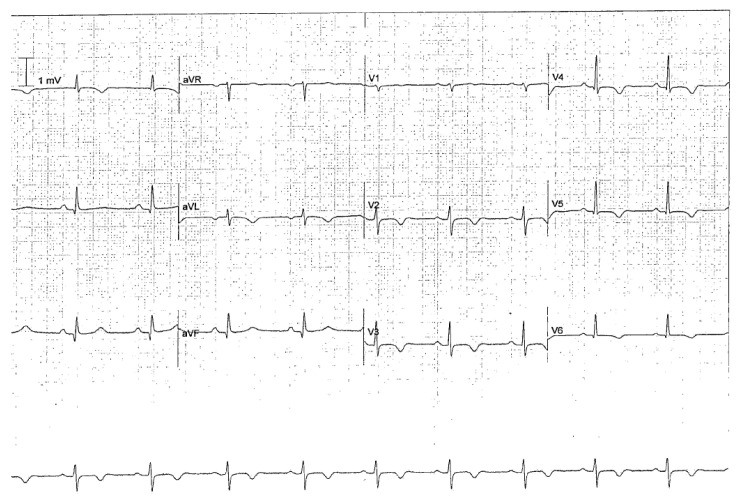
ECG in Takotsubo syndrome. A 62-year-old woman affected by focal somato-sensory epilepsy with tonic–clonic evolution secondary to previous hemorrhagic stroke in the right frontal lobe. The ECG control (12 h after a cluster of seizures) showed deep anterolateral T-wave inversion and a prolonged QTc interval (493 ms). Increased troponin (689 ng/L), an echocardiogram, and coronary angiographic images confirmed the diagnosis.

Seizure-induced TTS may occur immediately after the seizure and can be diagnosed at hospital admission. In about one-third of reported cases, the trigger is a tonic–clonic seizure. The diagnosis may be missed because of the absence of chest pain. Since it is not an uncommon finding and due to the risks associated with this cardiac dysfunction, an EKG is mandatory after a tonic–clonic seizure, especially in adult/old women (Figure 4).

### 3.4. Differential Diagnoses

Seizures and syncopes are often easily differentiated through medical history (see Table 2), even though in 20% of cases the diagnosis may remain equivocal and further investigations are needed. In some cases, homemade videos recorded with a smartphone can help with diagnosis, but in selected cases, the tilt test and/or video-polygraphic recordings are prescribed to allow a conclusive diagnosis.

**Figure 5:**
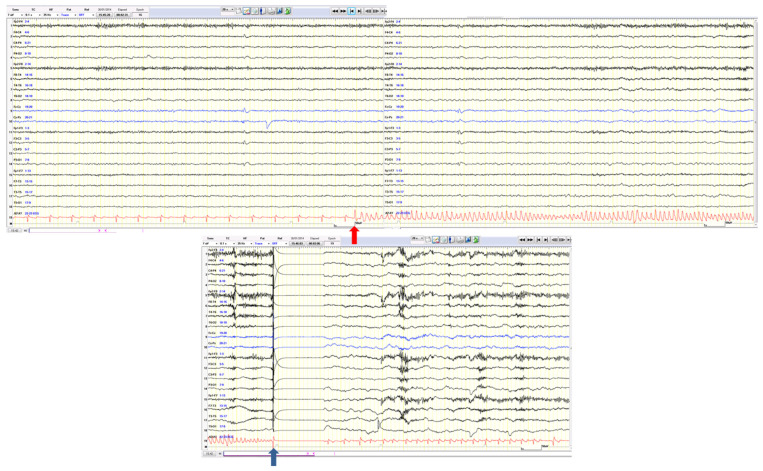
EEG–EKG video recording in Brugada syndrome. The patient’s symptomatology consisted of episodes of loss of consciousness preceded by tinnitus, internal heat, chest pain, and sweating. Witnesses objectified staring and blushing followed by diffuse stiffening with urinary incontinence. See EKG changes (red arrow) with the beginning of ventricular fibrillation. The EEG only shows a secondary progressive slowdown of the background activity to theta–delta activity. The blue arrow highlights pacemaker activation.

**Figure 6:**
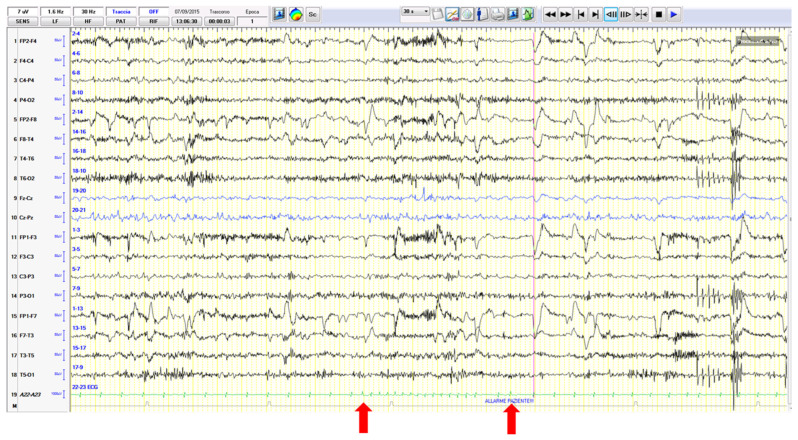
EEG–EKG video recording in the differential diagnosis between a focal seizure and arrhythmias. A middle-aged woman complained of daily episodes of “ascending epigastric sensation”. During these symptoms, brief supraventricular tachycardia episodes (red arrows) were recorded.

Apart from the differential diagnosis between syncopes and seizures and the relationships between seizures and syncopes in the same attacks as described above, another issue is that cardiac arrhythmias (such as paroxysmal sinus tachycardia, atrial fibrillation or flutter, and ventricular fibrillation) can generate symptoms similar to a focal seizure (seizure-mimic) which can be unraveled only by means of video-polygraphy (see two examples in Figure 5 and Figure 6). In these cases, the intriguing symptoms include epigastric discomfort, fear, and tachycardia.

## 4. Pathophysiology

Cardiovascular comorbidities are relatively common in people with epilepsy. While the most urgent issue in clinical practice may concern the differential diagnosis between seizures and syncopes or brief arrhythmic events, the cardiovascular consequences of seizures have been the object of many studies and debates, mostly to better understand the mechanisms of SUDEP. In this review, we have primarily described the cardiovascular effects of seizures and distinguished between ictal and post-ictal or interictal events. This distinction is of particular relevance since only post-ictal arrhythmias have been linked to SUDEP while ictal events are usually self-limiting.

Ictal tachycardia or bradycardia are the most frequent events. Interestingly, electrical stimulation of the human insular cortex suggested a hemispheric lateralization of the autonomic pathways, with the right hemisphere having a greater sympathetic influx and the left hemisphere having predominant parasympathetic control. More recent studies, however, have not confirmed the lateralization of autonomic control, probably because during epileptogenesis, networks are altered and remodeled and the onset zone is only a part of the structures activated during the seizure’s spread.

IA is the most intriguing cardiac effect of seizures and is associated with a definite clinical picture of syncope and a fall to the ground triggered by focal, usually temporal seizures. The precise mechanism of IA is unknown but is invariably self-limiting. It is likely the result of the diffusion of the epileptic activity to the structures of the central autonomic networks [29]. In fact, in humans, focal stimulation of different parts of the limbic system (i.e., amygdala and cingulate gyrus) may provoke asystole [30], suggesting that excitation of different gateways of the same system can produce the same result.

The role of sodium blocker antiepileptic drugs in increasing the risk of IA has been highlighted, and their withdrawal may contribute to resolving IA [31].

Additional contributing factors are the interictal cardiological and autonomic status of the patient as well as the cardiorespiratory and cardiovascular reflexes, which are known to be already chronically remodeled by seizures [32]. Alternatively, it has been hypothesized that seizure-induced fear and therefore catecholamine release [29] could evoke, in some patients, a vasovagal response, causing cardioinhibition and vasodilation. It has long been debated whether IA could be one of several potential mechanisms of SUDEP, which is the most common cause of death in longstanding uncontrolled epilepsy. Studies exploring the duration of seizures and asystole have demonstrated that IA causes a shorter duration of seizures and that the parasympathetic outflow and hypoperfusion mechanisms may contribute to terminating seizures and restoring normal cerebral excitability [33]. Moreover, no case of death has been reported so far in IA. Therefore, the role of IA in SUDEP appears unlikely. While syncopes mediated by IA may be induced by seizures, the reverse condition of syncopes causing seizures is exceptionally described in adults and may be caused by a transient anoxic insult as hypothesized for seizures occurring during an ischemic stroke. In contrast with IA, which is self-limiting and not associated with a risk of SUDEP, post-ictal asystole is less common, is associated with convulsive rather than focal (temporal lobe) seizures, and has a high fatality rate. All fatal cases reported in the Mortemus study [33] who had cardiorespiratory monitoring showed a consistent and previously unrecognized pattern after tonic–clonic seizures, characterized by post-ictal EEG suppression and transient or terminal cardiorespiratory dysfunction. The mechanism underlying this sequence of massive post-ictal EEG suppression, apnea, and terminal asystole has not yet been elucidated but does not involve the vaso-vagal activation pathways responsible for IA. Other post-ictal arrhythmias such as ventricular fibrillation are associated with a higher risk of SUDEP, likely to be mediated by the arrhythmogenic effects of convulsive seizures, by triggering the sympathetic nervous system, as reflected by the peak in catecholamines and electrodermal activity [34,35].

Arrhythmias in epilepsy may not only result from seizure activity but also from shared genetic susceptibility. Several genetic mutations of genes co-expressed in the brain as well as in the heart have been discovered, caused by means of alteration of cellular electrical function, both for seizures and cardiac comorbidity with an increased risk of SUDEP.

In particular, ion channels associated with long-QT syndrome or fatal arrhythmias (i.e., KCNQ1, KCNQ2, KCNH2, KCNJ2, KCNA1, SCN1A, SCN2A, SCN5A, SCN8A, SCN10A, CACNA1C, CACNA2D1, and RYR2) have been identified as possible common substrates of seizures and SUDEP [36,37]. Seizure activity may also cause transient myocardial ischemia and TTS. The latter represents a paradigmatic condition, showing the link between mental stress, cortical activation, and cardiac disease. Histological findings, including enlarged myocytes, cytoskeletal rearrangement, and damage of contractile proteins, support the pathogenic role of catecholamine overload in TTS.

## 5. Classification

**Figure 7:**
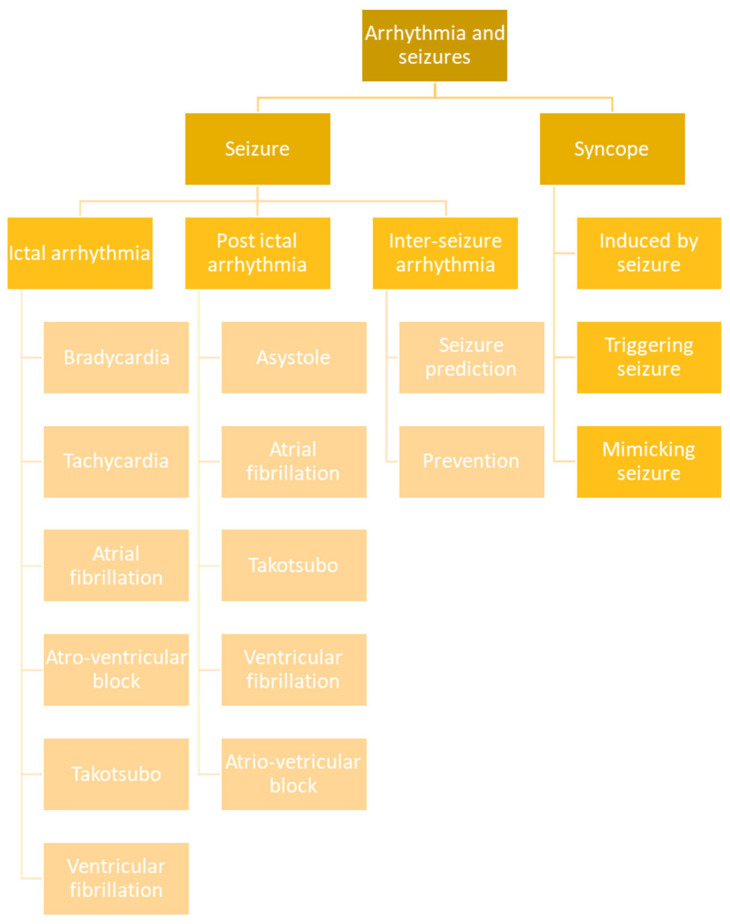
Schematic representation of possible correlations between seizures and arrhythmias.

On the basis of the above clinical and pathophysiological data, a classification of the arrhythmic events and syncopes connected to seizures may be proposed for clinical purposes (Figure 7).

In detail, we can distinguish ictal from interictal and post-ictal arrhythmic events, which may have completely different consequences and significance. Ictal arrhythmias include bradycardia, tachycardia, asystole, atrial fibrillation, ventricular fibrillation, and AV block. Post-ictal arrhythmias include asystole, atrial fibrillation, ventricular fibrillation, AV block, and Takotsubo syndrome. Except for atrial fibrillation, all of the above seizure-related cardiac events, when occurring in the post-ictal phase, are associated with a risk of SUDEP. However, higher risk is encountered with post-ictal asystole and post-ictal ventricular fibrillation, which are often referred to as near-SUDEP conditions. Interictal arrhythmias may be better termed with HRV analysis, which may have significance for seizure prediction and prevention. On the other hand, syncopes may be directly caused by an ictal discharge (not always mediated by IA) or, more rarely, may trigger a seizure. Sometimes the syncopes must be distinguished from seizures. The differential diagnosis may also include some arrhythmic events which can mimic focal seizures.

## 6. Conclusions

In conclusion, seizure-related cardiovascular changes are common in people with epilepsy.

Several mechanisms explain why these conditions tend to co-exist, including shared risk factors and the direct effects of seizures on autonomic functions or those resulting from their pharmacological treatment. Interestingly, interictal changes of HRV before seizures seem to predict the ictal event and are the basis for developing systems for the early detection of seizures. Seizure prediction is a crucial goal for many patients to avoid or limit the consequences of seizures. These are non-invasive tools based on continuous EKG, whose accuracy in detecting seizures could be increased in the future with other monitoring modalities including an accelerometer and electrodermal activity.

Further studies are needed to unravel the mechanisms of cardiovascular comorbidities and improve the risk profiling of patients.

## Figures and Tables

**Table 1:** International Takotsubo diagnostic criteria: ^a^ Wall motion abnormalities may remain for a prolonged period of time or documentation of recovery may not be possible. For example, death before evidence of recovery is captured. ^b^ Cardiac magnetic resonance imaging is recommended to exclude infectious myocarditis and for diagnostic confirmation of Takotsubo syndrome.

	International Takotsubo Diagnostic Criteria
1	Patients show transient ^a^ left ventricular dysfunction (hypokinesia, akinesia, or dyskinesia) presenting as apical ballooning or midventricular, basal, or focal wall motion abnormalities. Right ventricular involvement can be present. Besides these regional wall motion patterns, transitions between all types can exist. The regional wall motion abnormality usually extends beyond a single epicardial vascular distribution; however, rare cases can exist where the regional wall motion abnormality is present in the subtended myocardial territory of a single coronary artery (focal TTS). ^b^
2	An emotional, physical, or combined trigger can precede the Takotsubo syndrome event, but this is not obligatory.
3	Neurologic disorders (e.g., subarachnoid hemorrhage, stroke/transient ischemic attack, or seizures) as well as pheochromocytoma may serve as triggers for Takotsubo syndrome.
4	New ECG abnormalities are present (ST-segment elevation, ST-segment depression, T-wave inversion, and QTc prolongation); however, rare cases exist without any ECG changes.
5	Levels of cardiac biomarkers (troponin and creatine kinase) are moderately elevated in most cases; significant elevation of brain natriuretic peptide is common.
6	Significant coronary artery disease is not a contradiction in Takotsubo syndrome.
7	Patients have no evidence of infectious myocarditis. ^b^
8	Postmenopausal women are predominantly affected

**Table 2:** Differential diagnosis of seizures and syncopes.

	SYNCOPE	SEIZURE
TRIGGERS	Frequent	Rare
PRECEDING SYMPTOMS	Nausea, visual blurring, epigastric sensation, heat, headache, or tinnitus	Sensorial, psychic, somatosensory ‘auras’ ormotor phenomena
POSITION	Usually while standing or sitting. Supine very rare	Any
LOSS OF CONSCIOUSNESS	‘Fading away’ in young patients or abrupt loss in elderly persons	Abrupt loss
FALL	Slow or flaccid	Fast or tonic
SKIN COLOR	Pale	Sometimes perilabial cyanosis
EYE DEVIATION	Transient upward or lateral deviation	Sustained lateral deviation
INCONTINENCE	Common	Common
TONGUE BITE	Uncommon; localization: on the tip of the tongue	Common; localization: on the side of the tongue
CONVULSIONS	Lasts a few seconds and is arrhythmic, multifocal, or generalized	May last a few minutes and is rhythmic and generalized
DURATION	3–30 s	Depends on the type of seizure: up to 5 min for GTCS and shorter for others
POST-ICTAL PERIOD	Somnolence, fatigue, or headache	Confusion, somnolence, or headache

## Data Availability

The data are not publicly available due to ethical restrictions.

## Abbreviations

SUDEP: sudden unexpected death in epilepsy; HRV: heart rate variability; HR: heart rate; IA: ictal asystole; SEEG: stereoelectroencephalography; AV: atrioventricular; TTS: Takotsubo syndrome.
